# Dietary Consumption With Vitamin D Status Among Pregnant Women: A Descriptive-Analytic Study

**DOI:** 10.7759/cureus.50289

**Published:** 2023-12-10

**Authors:** Atiya Kareem Mohammed

**Affiliations:** 1 Department of Maternal-Neonatal Nursing, College of Nursing, University of Sulaimani, Sulaymaniyah, IRQ

**Keywords:** roche elecsys assay, nutritional needs, vitamin d status, pregnancy, dietary consumption

## Abstract

Aim: This study aimed to investigate the relationship between vitamin D status and diet between pregnant and non-pregnant women who attended a Maternity Teaching Hospital (MTH) in Sulaymaniyah, Iraq.

Materials and methods: A descriptive-analytic (cross-sectional) study was conducted at the MTH in Sulaymaniyah, Iraq. Data was collected from January to October 2022, including demographics, obstetric information, and dietary data. Vitamin D levels were measured using the Roche Elecsys assay (Roche Diagnostics, Indianapolis, Indiana, United States). The non-pregnant group consisted of women who were not pregnant at the time of this study, and the pregnant group had women with second trimester pregnancy at the time of enrollment.

Result: The study revealed a significant prevalence of vitamin D deficiency among pregnant women. In the study, pregnant participants (56.0% aged 30-39) and non-pregnant participants (54.3% aged 20-29) had similar ages (p>0.05). Both groups mostly had overweight individuals, with pregnant participants having a slightly higher mean body mass index (BMI). While vitamin D deficiency was more prevalent among pregnant women, the differences were not statistically significant. Notably, higher dairy intake was positively associated with higher vitamin D levels. Urban residency was common in both groups. Dietary habits were mostly similar, except for tea consumption (p<0.05), with non-tea drinkers having higher vitamin D levels. However, dietary patterns did not strongly correlate with vitamin D levels in the non-pregnant group.

Conclusion: This study reveals a significant prevalence of vitamin D deficiency among pregnant women, although the differences between pregnant and non-pregnant groups were not statistically significant. The positive association between higher dairy intake and increased vitamin D levels underscores the potential impact of dietary choices on vitamin D status during pregnancy. However, the study also suggests that dietary patterns alone may not strongly correlate with vitamin D levels in non-pregnant women. Overall, this highlights the importance of addressing vitamin D deficiency in pregnant women and underscores the need for personalized dietary guidance, taking into account individual preferences and habits to promote better maternal health.

## Introduction

Vitamin D is a fat-soluble vitamin first recognized for its role in preventing rickets. Vitamin D2 (ergocalciferol) and vitamin D3 (cholecalciferol) are the two main forms. Vitamin D2 is synthesized in plants, whereas vitamin D3 is produced in the skin when exposed to ultraviolet B (UVB) rays from sunlight via a precursor known as 7-dehydrocholesterol. Vitamin D obtained from dietary sources or sunlight exposure is converted into its active form in the body through a series of chemical reactions, primarily in the liver and kidneys. This conversion results in 1,25-dihydroxyvitamin D [1].

Optimal vitamin D supply is essential for fetal skeletal development during pregnancy. However, many researchers are interested in the non-classical action of vitamin D, such as enhancing insulin action, modulation of the immune system, and lung development. Also, studies showed that vitamin D deficiency might affect pregnancy by increasing the risk of preeclampsia and gestational diabetes mellitus (GDM) to the mother and small gestational age to the fetus [2].

Vitamin D can be obtained through dietary sources and exposure to sunlight, which triggers its synthesis in the skin. However, vitamin D synthesis is affected by seasons, time of day, exposure period, skin pigmentation, and latitude. Elderly individuals are at a higher risk of having low vitamin D levels as their skin synthesis decreases with age. To increase vitamin D intake, it is essential to consume vitamin D-rich foods, such as fatty fish, fish liver oils, and egg yolks, which are not commonly included in most people's diets. Vitamin D in meat and offal is typically low [3].

Research suggests that dairy consumption can positively impact vitamin D levels in pregnant women. A study published in Nutrients 2019 examined the association between dairy intake and vitamin D status in pregnant women. The study found that higher dairy consumption was associated with higher vitamin D levels in the blood [4].

A good source of vitamin D among foods, animal products have a higher amount of vitamin D. Fatty fish (salmon, tuna) and fish liver oil is the best dietary source of vitamin D, with a small amount in the egg yolk and animal liver. However, vegetables are an inferior source of vitamin D; therefore, vegetarians should get this vitamin through supplementation [5]. There is a significant difference in vitamin D metabolism between pregnant and non-pregnant states. This tremendous change in vitamin D metabolism has been known for about three decades but recently got more attention from researchers [6].

Any link between vitamin D deficiency and the development of pregnancy complications in Sulaymaniyah, Iraq, will help reduce the chances of getting pregnancy complications. Understanding the underlying causes is one of the most critical aspects of addressing community health issues. Most of these complications can be avoided by examining the serum vitamin D levels in preconception counselling programs.

This study aims to demonstrate the association between vitamin D deficiency and the prevalence of vitamin D insufficiency and vitamin D deficiency between pregnant and non-pregnant women in Sulaymaniyah, Iraq. Moreover, the study investigated dietary factors that may affect the serum vitamin D level.

## Materials and methods

A descriptive-analytic (cross-sectional) study was conducted at the Maternity Teaching Hospital (MTH) in Sulaymaniyah, Iraq. Purposive (non-probability) sampling was employed to select participants from women visiting the hospital during the study period. Patients aged less than 39 years, primiparous and multipara, and pregnant women in the second trimester were included, while those with complications and recent vitamin D supplement use were excluded. The non-pregnant group consisted of women who were not pregnant at the time of this study, and the pregnant group had women with second trimester pregnancy at the time of enrollment.

Data collection took place from January 3 to October 1, 2022, involving 262 eligible women. Anthropometric measurements were taken, including height and weight, to calculate body mass index (BMI) as an indicator of obesity risk. Data collection consisted of direct interviews, blood sample processing, and serum storage at -20°C for later vitamin D level measurements.

The study found that 25-hydroxyvitamin D (25(OH)D), a form of vitamin D, remains stable for different durations at varying temperatures, suggesting that serum and plasma can be used for vitamin D level measurement. The questionnaire covered socio-demographic characteristics, obstetric history, dietary habits (including vitamin D sources), and serum vitamin D status, categorized as deficiency (less than 20 ng/mL), insufficiency (20-30 ng/mL), and optimal (above 30 ng/mL) [7].

Statistical analysis was conducted using IBM SPSS Statistics for Windows, Version 22.0 (Released 2013; IBM Corp., Armonk, New York, United States) and Microsoft Excel (Microsoft Corporation, Redmond, Washington, United States). Results were presented in frequencies, percentages, and mean standard deviation (SD), with appropriate statistical tests applied. Significance levels were set at p≤0.05, with <0.001 considered highly significant. To assess the association between categorical variables and pregnancy status (pregnant versus non-pregnant), a chi-squared test was applied. 

The research protocol, reviewed by the Scientific and Ethics Committee of the University of Sulaimani's College of Medicine (approval number 129), prioritized participant confidentiality and anonymity. Ethical committee approval was obtained on June 8, 2022, with oversight from the MTH. Verbal consent was obtained from all participants, and strict measures were implemented to safeguard data confidentiality and participant anonymity throughout the study.

## Results

Table 1 presents a demographic comparison between pregnant and non-pregnant groups.

**Table 1:** Differences between pregnant and non-pregnant groups according to socio-demographic characteristics *: highly significant; SD: standard deviation

Variables	Items	Pregnant group frequency (%) (n=100)	Non-pregnant group frequency (%) (n=162)	p-value F-test
Age (in years)	Less than 20 years	3 (3%)	6 (3.70%)	0.139
	20-29 years	41 (41%)	88 (54.32%)	
	30-39 years	56 (56%)	68 (41.97%)	
	Mean±SD	30.10±4.95	28.33±5.03	
Body mass index	Underweight	1 (1%)	11 (6.8%)	0.038
	Normal	34 (34%)	43 (26.5%)	
	Overweight	47 (47%)	70 (43.2%)	
	Obese	11 (11%)	32 (19.8%)	
	Morbid obese	7 (7%)	6 (3.7%)	
Education	Illiterate	5 (5%)	1 (0.6%)	0.000*
	Primary school graduate	26 (26%)	30 (18.5%)	
	Secondary school graduate	36 (36%)	86 (53.1%)	
	High education	33 (33%)	45 (27.8%)	
Residency	Urban	63 (63%)	155 (95.7%)	0.000*
	Suburban	33 (33%)	5 (3.1%)	
	Rural	4 (4%)	2 (1.2%)	
Occupation	Employee	25 (25%)	33 (20.4%)	0.381
	Non-employee	75 (75%)	129 (79.6%)	

Pregnant participants tended to be older, with 56.0% aged 30-39, while 54.3% of non-pregnant participants were aged 20-29. The groups had no significant age difference (p=0.139). In terms of BMI, both groups had a majority of overweight individuals, but the mean BMI differed significantly (p=0.038), with pregnant participants slightly higher. Most in both groups were non-employed (75.0% pregnant, 79.6% non-pregnant) with no statistical difference (p=0.381). Education varied significantly (p=0.000), with more postgraduates in the pregnant group (32%) compared to the non-pregnant group (27.2%). Residency patterns also differed significantly, with more urban dwellers in both groups (p=0.000). Dress style preferences were similar (p=0.465) in both groups, with most opting for full coverage.

Table 2 reveals the obstetric details of the study population.

**Table 2:** Differences in pregnant and non-pregnant groups based on obstetric history

Variables	Items	Pregnant group frequency (%) (n=100)	Non-pregnant group frequency (%) (n=162)	p-value
Gravida	Primigravida	23 (23%)	61 (37.7%)	0.014
	Multigravida	77 (77%)	101 (62.3%)	
Para	Primiparous	68 (68%)	138 (85.2%)	0.001
	Multipara	32 (32%)	24 (14.8%)	

In the pregnant group, 23.0% were primigravida, compared to 37.7% in the non-pregnant group, showing a significant difference in gravidity (p=0.014). In terms of parity, 68.0% of the pregnant group and 85.2% of the non-pregnant group were primiparous, while 32.0% of the pregnant group and 14.8% of the non-pregnant group were multiparas, with a significant difference (p=0.001).

Table 3 outlines the dietary habits in both pregnant and non-pregnant groups.

**Table 3:** Differences between pregnant and non-pregnant groups according to their dietary history **: regular milk consumption (cow's milk)

Variables	Items	Pregnant group frequency (%) (n=100)	Non-pregnant group frequency (%) (n=162)	p-value
					Fish consumption	Non	27 (27%)	45 (27.8%)	0.078
						Once a month	23 (23%)	55 (34%)	
Every two weeks	31 (31%)	31 (19.1%)							
Once a week	15 (15%)	20 (12.3%)							
Twice a week	2 (2%)	10 (6.2%)							
More than twice a week	2 (2%)	1 (0.6%)							
Milk consumption	Yes**	48 (48%)	101 (62.3%)	0.023
	No	52 (52%)	61 (37.7%)	
	Low fat	13 (27.1%)	24 (23.8%)	0.359
	Soya	0 (0%)	4 (4.0%)	
	Full-fat milk	35 (72.9%)	73 (72.3%)	
How many cups of milk per day	1 cup	35 (74.5%)	65 (64.4%)	0.147
	2 cups	8 (17.1%)	22 (21.8%)	
	3 cups	4 (8.4%)	9 (8.8%)	
	4 cups	0 (0.0%)	5 (5.0%)	
Tea consumption	Yes	65 (65%)	108 (66.7%)	0.442
	No	35 (35%)	54 (33.3%)	
Cups of tea per day	Less than 4	62 (91.2%)	69 (88.5%)	0.207
	5-8	6 (8.8%)	4 (5.1%)	
	More than 8	0 (0.0%)	5 (6.4%)	
Cheese consumption per week	Less than 5 portions	38 (66.7%)	41 (56.9%)	0.151
	5-10 portions	16 (28.1%)	25 (34.7%)	
	11-15 portions	3 (5.2%)	2 (2.7%)	
	More than 15 portions	0 (0.0%)	4 (5.7%)	
Yogurt consumption per week in portion	Less than 5	25 (33.7%)	39 (46.4%)	0.661
	5-10	47 (63.5%)	37 (44.1%)	
	More than 10	2 (2.8%)	8 (9.5%)	
Coffee consumption per week in cups	1 cup	8 (72.7%)	18 (90.0%)	0.503
	2 cups	3 (27.3%)	1 (5.0%)	
	3 and more	0 (0.0%)	1 (5.0%)	

Fish consumption was most frequent every two weeks in the pregnant group (31.0%) and once a month in the non-pregnant group (34.0%), with no significant difference (p=0.076). Regarding milk, differences were significant (p=0.023) with full-fat milk being preferred in both groups. Tea consumption was regular in about 65% of participants in both groups, with no significant difference (p=0.207). Cheese consumption was generally below five portions weekly in both groups, without significant variation (p=0.151). Yogurt consumption ranged from five to 10 portions weekly in 63.5% of the pregnant group and less than five portions in 46.4% of the non-pregnant group, with no significant difference (p=0.661). Coffee consumption was mostly one cup in both groups (72.7% pregnant, 90% non-pregnant), with no significant variation (p=0.503). Overall, dietary patterns were largely similar between the two groups.

In Table 4, the study explored the link between dietary habits and vitamin D levels in the non-pregnant group.

**Table 4:** Vitamin D status concerning dietary history in the non-pregnant group ng/mL: nanograms per milliliter; SD: standard deviation

Variables	Vitamin D (ng/mL) mean±SD	p-value	Significance
Fish consumption			
Non	17.54±15.2	0.92	No significance
Once a month	18.72±15.5		
Every two weeks	17.18±16.2		
Once a week	14.37±10.7		
Twice a week	19.58±14.7		
More than twice a week	17.70±0.00		
Milk consumption			
				Yes	18.02±13.5	0.653	No significance
				No	16.93±16.89		
Type of milk			
				Low fat	17.33±9.56	0.254	No significance
Soya	7.42±4.55						
Full-fat milk	18.02±14.77						
How many cups of milk per day			
1 cup	19.32±14.88	0.321	No significance
2 cups	13.29±7.91		
3 cups	20.31±16.29		
4 cups	17.78±5.64		
Drink tea			
Yes	15.99±13.53	0.042	Significant
No	20.84±15.28		
Strong tea			
				Yes	12.21± 5.71	0.541	No significance
				No	16.12±16.23		
Tea consumption per day in cup			
Less than 4	16.18±16.4	0.372	No significance
4-8	6.48±3.31		
More than 8	10.12±7.23		
Cheese consumption per week			
Less than 5 portions	17.98±16.5	0.681	No significance
5-10 portions	16.24±19.1		
11-15 portions	17.50±19.1		
More than 15 portions	7.02±6.23		
Yogurt consumption per week			
Less than 5 portions	13.57±10.6	0.38	No significance
5-10 portions	18.84±21.1		
More than 10 portions	10.25±3.86		
Coffee consumption per week			
1 cup	20.26±23.2	0.821	No significance
2 cups	7.46±0.0		
3 cups and more	11.80±0.0		

The findings showed no statistically significant difference in vitamin D levels based on fish consumption, although those who consumed fish twice weekly had a slightly higher mean vitamin D level. Similarly, there was no significant difference in vitamin D levels between milk consumers and non-consumers, irrespective of milk type or daily quantity. Regular tea consumption did result in a statistically significant difference, with non-tea drinkers having higher mean vitamin D levels. However, the type of tea (strong or regular) did not affect vitamin D levels. Cheese intake did not yield significant differences in vitamin D levels, while coffee consumption also had no significant impact. Overall, dietary habits did not strongly correlate with vitamin D levels in this non-pregnant group, except for tea consumption.

Table 5 presents the relationship between vitamin D levels and dietary habits during pregnancy.

**Table 5:** Vitamin D status concerning dietary habits in the pregnant group ng/mL: nanograms per milliliter; SD: standard deviation

Variables	Vitamin D (ng/mL) mean±SD	p-value
Fish consumption		
Non	13.15±9.30	0.013
Once a month	10.40±6.71	
Every two weeks	15.60±13.31	
Once a week	20.22±23.56	
Twice a week	44.00±12.72	
More than twice a week	13.00±5.65	
Milk consumption		
Yes	15.15±16.39	0.892
No	14.77±11.35	
Type of milk		
Low fat	10.22±7.19	0.208
Soya	0.0±0.0	
Full-fat milk	16.98±18.45	
How many cups of milk per day		
1 cup	19.32±14.88	0.321
2 cups	13.29±7.91	
3 cups	20.31±16.29	
4 cups	17.78±5.64	
Drink tea		
Yes	14.01±10.60	0.351
No	16.77±18.85	
Strong tea		
Yes	14.55±11.03	0.494
No	12.26±9.21	
Tea consumption per day in cup		
Less than 4	14.42±10.8	0.444
5-8	10.89±9.20	
More than 8	0.0±0.00	
Cheese consumption per week		
Less than 5 portions	16.59±18.9	0.677
5-10 portions	13.33±9.46	
11-15 portions	10.10±5.40	
More than 15 portions	0.0±0.00	
Yogurt consumption per week		
Less than 5 portions	11.12±8.4	0.094
5-10 portions	18.93±17.7	
More than 10 portions	14.65±3.32	
Coffee consumption per week		
1 cup	12.32±12.2	0.786
2 cups	14.52±10.1	
3 and more	0.0±0.0	

A significant difference was found in vitamin D levels based on fish consumption frequency. Those consuming fish twice weekly had a higher mean vitamin D level. However, milk intake did not yield a significant difference, although regular milk consumers had a slightly higher mean vitamin D level. The type of milk or daily quantity consumed did not affect vitamin D levels. Tea consumption did not significantly impact vitamin D levels, and the same applied to strong tea intake. Cheese and yogurt consumption also did not yield statistically significant differences in vitamin D levels, though those with lower cheese intake had a slightly higher mean level and those with moderate yogurt consumption had a slightly higher mean vitamin D level. Coffee intake did not significantly affect vitamin D levels. Overall, fish consumption had the most notable impact on vitamin D levels during pregnancy.

Table 6 reveals no significant differences in serum vitamin D levels between pregnant and non-pregnant groups (p=0.263).

**Table 6:** Comparison between the vitamin D status of the pregnant group and non-pregnant group

Vitamin D class	Pregnant group (n=100) frequency (%)	Non-pregnant group (n=162) frequency (%)	p-value
Deficient (20 ng/mL)	77 (77%)	111 (68.5%)	0.263
Insufficient (20-30 ng/mL)	13 (13%)	33 (20.4%)	
Sufficient (30 ng/mL)	10 (10%)	18 (11.1%)	

However, vitamin D deficiency was more prevalent in the pregnant group (77%) than the non-pregnant group (68.5%), while vitamin D sufficiency was similar.

## Discussion

This study compared the vitamin D levels between pregnant women and non-pregnant women in Sulaymaniyah, Iraq. Also, this study examined the proposed associated risk factors for vitamin D deficiency. Socio-demographic characteristics and several obstetric and medical histories could, in different pathways, increase the risk of vitamin D deficiency. In total, 262 pregnant women were recruited in the current study; 100 were with GDM, and 162 were not pregnant.

The current study investigated some dietary variables, including consumption of milk, yogurt, cheese, tea, and coffee, concerning vitamin D and pregnancy. The only significant difference that has been found between the two groups was milk consumption, in which higher numbers of non-pregnant participants had regular milk consumption. In one study, milk consumption habits of pregnant women were studied to find their association with the risk of vitamin D deficiency in China. They carried out a large cohort study of women in their middle age, and they found that consumption of calcium from dairy products was associated with a lower risk of vitamin D deficiency [8].

Schoenaker et al. did a large population-based prospective cohort study, which collected 3,853 women from 2003 to 2012, examining the association between dietary patterns and the risk of vitamin D deficiency. They found no association between low-fat dairy consumption and the risk of vitamin D deficiency [9].

Tea is one of the most popular beverages in the world, following water. In the Kurdistan region, tea is widely consumed by all age groups, especially adults; however, no study has been carried out concerning the potential effect of tea consumption on human metabolism. Black tea is mostly consumed in this region and is mainly served with sugar cubes. Some components in the tea, such as polyphenols, are proposed to be protective factors in the development of several chronic diseases, such as cardiovascular diseases and diabetes. Some evidence also showed that the components in the tea might affect glucose metabolism and insulin signaling [10]. The association between tea consumption and the risk of vitamin D deficiency was investigated in the study by Hinkle et al. [11]. They conducted a large cohort study to find the relationship between tea and coffee consumption and the risk of vitamin D deficiency among pregnant women. They reported that the risk of vitamin D deficiency was not consistently or significantly increased with tea consumption.

In 2007, Adeney and colleagues looked at the association between coffee consumption and the risk of vitamin D deficiency development in pregnant women for the first time [12]. They observed that the risk of vitamin D deficiency was reduced among pregnant women who consumed a moderate amount of caffeinated coffee. Many factors may contribute to the differences in the results of the studies, for instance, differences in the study population, exposure, and exposure measurement.

In this study, coffee consumption was measured using cups per week. The size of the cups, the amount of coffee in each cup, and the methods used to prepare the coffee may vary. Also, the studies did not distinguish the different tea strengths and decaffeinated coffee or type of coffee.

Low serum 25(OH)D levels continue to be a problem worldwide. While some in vitro studies show a caffeine-induced decrease in vitamin D receptor expression, more research is needed to determine the extent of caffeine consumption and its effects on 25(OH)D levels [13]. This study has found no significant differences between vitamin D levels of the women with different amounts of milk consumption in both groups.

Among commonly eaten food items, small numbers contain vitamin D. The current study looked at some items such as dairy products, including milk, yogurt, cheese, and fish. Natural milk contains negligible vitamin D; however, manufactured milk's vitamin D content varies according to the manufacturer's guidelines. A study conducted in Saudi Arabia investigated the frequency of milk consumption and its relation with vitamin D levels across various age groups. Frequent milk consumption was associated with higher vitamin D levels [14]. The commonly consumed milk in Sulaymaniyah, Iraq, is fortified with vitamin D, but the quantity is low, for instance, Almarai (Riyadh, Saudi Arabia) contains 54.6 IU of vitamin D3, and kale has no vitamin D.

Food frequency questionnaire and seven- or three-day dietary recall are examples of the methods used by most studies concerning the dietary habits of the specific population. However, in the current study, due to the abysmal compliance of the study population, using a food diary seems to be not a reliable method to collect data regarding dietary habits. The association between vitamin D levels and the consumption of fortified milk with vitamin D was examined among pregnant women in two studies. They reported that consuming fortified milk with multivitamins increases the serum level of vitamin D. The mean of milk consumption was one serving per day. They reported that vitamin D deficiency is common among women who do not regularly drink milk [15].

The level of the umbilical cord blood (UCB) vitamin D status was studied compared to the dietary history of pregnant women [16]. The study reported that intake of fortified dairy products was positively associated with maternal and UCB 25(OH)D. A large systematic review and meta-analysis were carried out to illustrate the association between the consumption of milk fortified with vitamin D and serum vitamin D levels between 1993 and 2017. A positive association was reported between consuming fortified milk with vitamin D and serum vitamin D concentration. Several countries like Canada, Finland, Ireland, Australia, Norway, Spain, Sweden, and the United Kingdom legally must fortify milk with vitamin D [17].

Consuming fish twice weekly significantly increases serum vitamin D levels, potentially protecting against deficiency. A meta-analysis of randomized controlled trials in 2014 found that fish consumption, especially fatty fish like salmon, can raise vitamin D levels by an average of 4.4 nmol/L [18]. For optimal results, 300 grams of fish twice a week for less than four weeks is recommended. Wild salmon contains approximately 988 IU of vitamin D3 per serving, while farmed salmon has 25% of that amount [19].

The results of the studies described previously show that vitamin D levels were higher in subjects that consumed fish; however, it cannot optimize vitamin D status completely. On the other hand, it should be considered that a high fish intake is associated with another side effect because of the accumulation of environmental pollutants. In this term, supplements or fortified food need to be used to increase vitamin D concentration sufficiently without UVB.

This study compared serum 25(OH)D levels in pregnant and non-pregnant women. It found no significant differences in vitamin D levels between the groups. However, vitamin D deficiency was more prevalent in pregnant women. The study suggests that dietary sources of vitamins D2 (from vegetables) and D3 (from animal products like fish) partially meet human vitamin D needs [20]. Vitamin D is crucial for pregnant women and newborns. During pregnancy, vitamin D metabolism changes significantly, with a twofold increase in calcitriol during the first trimester, a 25% decrease in UCB 25(OH)D levels, and an increased expression of vitamin D receptors and metabolic enzymes in the placenta. Maternal calcitriol and vitamin D binding protein (VDBP) levels rise, impacting neonatal vitamin D stores [21].

In cases where dietary intake or sun exposure is insufficient for maintaining adequate vitamin D levels, healthcare providers may recommend vitamin D supplementation during pregnancy to ensure optimal vitamin D status for both the mother and the developing fetus.

The study limitations are the study focused solely on pregnant women attending a specific maternity hospital, which may not be representative of all pregnant women in the general population. This could introduce selection bias. The reliance on self-reported dietary data is another notable limitation. Furthermore, the study's sample size of 262 pregnant women may be considered relatively small. BMI, calculated based on pre-pregnancy weight or even weight gain during pregnancy, may not accurately reflect an individual's health status during this unique period. Pregnancy involves dynamic changes in body composition, including increased blood volume, placental and fetal growth, and changes in fat distribution, which can affect BMI calculations. A larger sample size would provide more statistical power, enhance the precision of the estimates, and increase the generalizability of the findings. Conducting intervention studies that assess the impact of dietary modifications, supplementation, or educational programs on vitamin D status in pregnant women can provide insights into practical strategies for improving vitamin D levels during pregnancy. This knowledge can inform healthcare practices and policies aimed at optimizing the nutritional well-being of pregnant women and, by extension, the health of their offspring.

## Conclusions

The history of vitamin D deficiency was significantly higher among pregnant women. In the group of pregnant women, the mean of vitamin D was significantly higher in women who ate fish twice a week, did regular exercise, did not require dental treatment, were aged between 29 and 39 years, and had a high socioeconomic class. In the group of non-pregnant women, the vitamin D level was significantly higher among the women who did not drink tea and had partly covered clothes style.
